# Improved individual hand hygiene compliance with a multimodal hand hygiene intervention – the results of the PROHIBIT (Prevention of Hospital Infections By Intervention and Training) project

**DOI:** 10.1186/2047-2994-4-S1-P147

**Published:** 2015-06-16

**Authors:** T Van Der Kooi, H Boshuizen, S de Greeff, H Grundmann, W Zingg

**Affiliations:** 1 CIb, Bilthoven, Netherlands; 2 SIM, RIVM, Bilthoven, Netherlands; 3 Med Microbiology, University Medical Center of Groningen, Groningen, Netherlands; 4 ICP, University Hospitals of Geneva, Geneva, Switzerland

## Introduction

The PROHIBIT study aimed at inventorying and analysing national and local infection prevention activities in Europe and to test two interventions of proven efficacy in central venous catheter (CVC) bloodstream infection (CRBSI) reduction: a multimodal CVC bundle and a multimodal hand hygiene (HH) improvement strategy.

## Objectives

Analyze the contribution of individual health care workers’ (HCW) HH compliance to the overall increase in HH.

## Methods

Intensive Care Units of 14 hospitals in 11 European countries participated in this prospective stepped wedge cluster-randomized trial. HH was evaluated by direct observation conform the World Health Organization. Ten centres collected individual HH compliance data of HCW. Generalized linear mixed modelling was used, of HH compliance (%) per observation session.

## Results

In 9762 sessions 46,729 HH opportunities of 1874 HCWs were collected in the 10 centres. Seven of the 10 centres (7980 sessions) were allocated to implement the HH campaign, alone or with the CVC strategy. Average baseline compliance in these centres was 43.1%, which increased to 60.8% after the start of the intervention. The proportion of HCWs with 0% compliance decreased from 26% during baseline to 11% after the implementation of the HH campaign whereas the proportion complying 100% doubled from 16% to 33%. Many HCWs were observed in <4 sessions only (34.4%) and not all were assessed during both periods. Individual changes in HH and the variance among HCWs were evaluated in HCWs assessed in both periods, with ≥ 4 observed sessions (5406) as HCWs with <4 sessions inflate the variance. Of these 375 HCWs 70.4% increased HH >10%, 11.5% remained constant (±10%) and 16.5% decreased >10%. The variance among HCWs within hospitals when comparing both periods decreased (p=0.36). This implies that the difference between relatively poor and good compliers remained comparable.

## Conclusion

The multimodal HH campaign in our multicentre study resulted in the significant increase of the average HH compliance. The HH improvement was due to behaviour change of the individual HCWs.

## Disclosure of interest

None declared.

